# A comprehensive review about immune responses and exhaustion during coronavirus disease (COVID-19)

**DOI:** 10.1186/s12964-022-00856-w

**Published:** 2022-06-02

**Authors:** Rebar N. Mohammed, Rozita Tamjidifar, Heshu Sulaiman Rahman, Ali Adili, Shadi Ghoreishizadeh, Hossein Saeedi, Lakshmi Thangavelu, Navid Shomali, Ramin Aslaminabad, Faroogh Marofi, Mina Tahavvori, Svetlana Danishna, Morteza Akbari, Gülinnaz Ercan

**Affiliations:** 1 Medical Laboratory Analysis Department, College of Health Sciences, Cihlan University of Sulaimaniya, Kurdistan Region, Iraq; 2 College of Veterinary Medicine, University of Sulaimani, Sulaimaniyah, Iraq; 3 I.M. Sechenov First Moscow State Medical University, Moscow, Russia; 4 Department of Physiology, College of Medicine, University of Sulaimani, Sulaimaniyah, Iraq; 5 Department of Medical Laboratory Sciences, Komar University of Science and Technology, Sarchinar District, Sulaimaniyah, Iraq; 6 Department of Oncology, Tabriz University of Medical Sciences, Tabriz, Iran; 7 Immunology Research Center, Tabriz University of Medical Sciences, Tabriz, Iran; 8 Department of Pharmacology, Saveetha Dental College, Saveetha Institute of Medical and Technical Science, Saveetha University, Chennai, India; 9 Department of Medical Biochemistry, Faculty of Medicine, Ege University, 35100 Izmir, Turkey; 10 Department of Stem Cell, Institute of Health Sciences, Ege University, Izmir, Turkey

**Keywords:** SARS-CoV-2, COVID-19, Immune system exhaustion, T cells

## Abstract

Coronavirus disease (COVID-19) is a viral infectious disease caused by the severe acute respiratory syndrome coronavirus 2 (SARS-CoV-2) virus. The infection was reported in Wuhan, China, in late December 2019 and has become a major global concern due to severe respiratory infections and high transmission rates. Evidence suggests that the strong interaction between SARS-CoV-2 and patients' immune systems leads to various clinical symptoms of COVID-19. Although the adaptive immune responses are essential for eliminating SARS-CoV-2, the innate immune system may, in some cases, cause the infection to progress. The cytotoxic CD8^+^ T cells in adaptive immune responses demonstrated functional exhaustion through upregulation of exhaustion markers. In this regard, humoral immune responses play an essential role in combat SARS-CoV-2 because SARS-CoV-2 restricts antigen presentation through downregulation of MHC class I and II molecules that lead to the inhibition of T cell-mediated immune response responses. This review summarizes the exact pathogenesis of SARS-CoV-2 and the alteration of the immune response during SARS-CoV-2 infection. In addition, we’ve explained the exhaustion of the immune system during SARS-CoV-2 and the potential immunomodulation approach to overcome this phenomenon.

**Video Abstract**

**Supplementary Information:**

The online version contains supplementary material available at 10.1186/s12964-022-00856-w.

## Background

Severe acute respiratory syndrome coronavirus 2 (SARS-CoV-2) is caused by the coronavirus (COVID-19), a common human-animal pathogen that causes severe damage to the human respiratory system. COVID-19 spread rapidly in Wuhan, China, in late December 2019 and has become a primary global concern. This infection causes severe clinical symptoms in patients with COVID-19, such as acute respiratory distress, hypoxemia, dyspnea, cytokine release syndrome, and lymphopenia [1, 2]. The piece of evidence suggested that homeostasis of the innate and adaptive immune system plays a critical role in developing pneumonia and other clinical symptoms in patients with COVID-19 [3].

SARS-CoV-2 is a linear positive-sense (+) single-stranded RNA (ssRNA) virus and a significant member of the large coronaviruses group (4). Interaction of SARS-CoV-2 with the host cells occurs via attachment of viruses spike (S) protein to the host angiotensin-converting enzyme 2 (ACE2) receptor, processed by transmembrane protease serine 2 (TMPRSS2). TMPRSS2 is a critical fusion peptide for fusing the virus into the host cells [4]. SARS-CoV-2 and other coronaviruses encode several non-structural manipulating virulence proteins that interact with the host immune system, causing alteration of host cells' physiology [5]. Although the interaction between SARS-CoV-2 with the host immune system, underlying molecular immune mechanisms, and the cause of the difference in clinical symptoms have been studying since the virus identification, the means by which the virus causes immune evasion and exhaustion has not entirely been elucidated [6]. Interaction between SARS-CoV-2 and the host immune system is one of the significant causes of diversity in clinical signs of COVID-19 so that some individuals with COVID-19 remain asymptomatic. In contrast, others indicated severe clinical complications, such as acute respiratory distress and pneumonia [7, 8].

SARS-CoV-2 stimulates several host immune responses such as increased synthesis of interferons type I (IFNs), induction and maturation of dendritic cells (DCs), and increased secretion of inflammatory factors, which are essential in limiting the spread of viruses [9]. In addition, SARS-CoV-2 causes activation of both innate and acquired immune responses. CD8+ T cells directly kill infected CD4+ T cells as well as stimulate B cells to secret virus-specific antibodies such as immunoglobulin G (IgG) and immunoglobulin M (IgM). T helper cells secrete various mediators such as pro-inflammatory cytokines to amplify immune response and help other immune cells. In addition, the secretion of antibodies and complement factors (C3a and C5a) by host immune cells is critical in the antiviral immune responses. On the other hand, SARS-CoV-2 can block the host immune response by suppressing T cells by inducing apoptosis. However, the underlying pathogenesis of SARS-CoV-2 in the host immune system is less known [10–12].

Further recognition of SARS-CoV-2 interaction with the host immune system is required to develop a novel therapeutic approach against COVID-19. This review summarises pathogenesis and immunological features of SARS-CoV-2 and alteration of the host immune system by SARS-CoV-2. Moreover, we discussed the exhaustion of the host immune system by SARS-CoV-2 and the potential immunomodulation approach to overcome immune exhaustion.

## Immune response to SARS-CoV-2

### Innate immune response to SARS-CoV-2

The innate immune response is the first line for immunological combat against SARS-CoV-2. SARS-CoV-2 enters the host cell through cathepsin L (CTSL) or endocytosis following binding of SARS-CoV-2 spike (S) protein to angiotensin-converting enzyme 2 (ACE2), which is completed by transmembrane serine protease 2 (TMPRSS2) or TMPRSS2-dependent direct fusion [13, 14]. After viral infection, pathogen-associated molecular patterns (PAMPs) and damage-associated molecular patterns (DAMPs), viral small molecular motifs, are recognized by toll-like receptors (TLRs) or pattern recognition receptors (PRRs). Subsequently, intracellular signaling cascades such as interferon regulatory factors (IRFs) and nuclear factor-kappa B (NF-κB) are activated. These events ultimately cause the production of pro-inflammatory cytokines and type I interferons (IFNs) [15, 16]. Typically, IFN induces apoptosis induction to protect host cells from viral spread and provide an anti-viral immune response limiting viral replication in infected host cells. However, SARS-CoV-2, by expressing several factors, such as open reading frame 6 (ORF6), suppresses the production of anti-viral IFN-I response [17, 18]. The initial delay of IFN-I response along with virus spreading in host cells leads to disease progression and exacerbates inflammation. Therefore, IFN responses may play an important role in heterogeneous disease severity in patients with COVID-19 [3, 19].

The complement system is another component of the innate immune response against SARS-CoV-2, a rapid immune surveillance system that bridges innate and adaptive immune responses [20]. In patients with COVID-19, an excessive complement system activation has been shown to be associated with intravascular coagulation, endothelial cell dysfunction, and chronic and acute inflammation [21]. Strong systemic or local activation of the complement system in patients with COVID- 19 provides a logical explanation for complement system inhibition as a therapeutic approach in COVID-19 [22, 23].

**Fig. 1:**
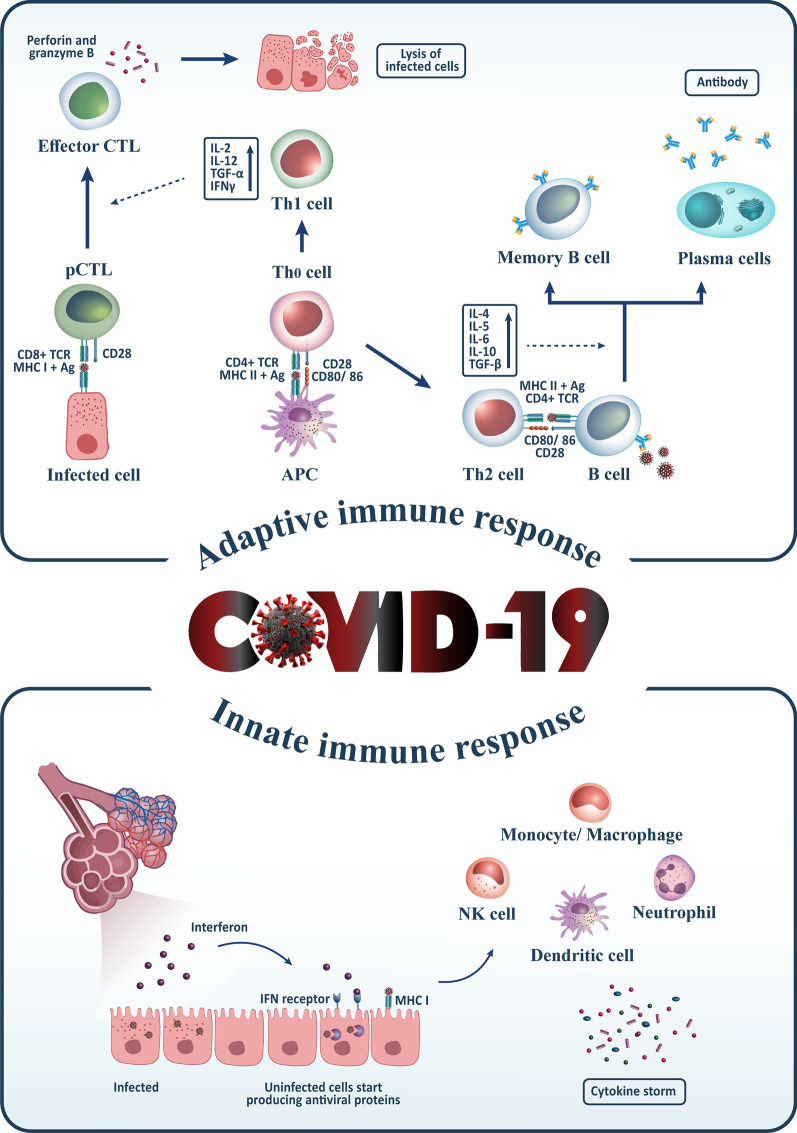
The innate and acquired immune responses against COVID-19. In the natural immune system, dendritic cells, monocytes/macrophages, neutrophils, and the production of Type 1 interferons come across against the COVID-19 virus. On the other hand, in the adaptive immune system, B cells produce antibodies and T cells by differentiating to cytotoxic T lymphocytes (CD8+) or helper T cells (CD4+) interacting with the COVID-19 infection. pTLs (primary cytotoxic T lymphocytes) are activated by binding to MHC-1 on infected cells, differentiate to effector CTL and lysis infected cells by producing perforin and Granzyme. Besides, T helper (Th) 1 cell can induce differentiation of pCTL to effector CTL by producing IL-2, IL-12, TGF-β, and IFN-γ, as well as Th2 cells, help B cells to produce antibodies by secreting IL-4, 5, 6, 10, and TGF-β

Interaction of innate immune system and coagulation system (a process called immunothrombosis) is dysregulated in patients with severe COVID-19, causing localized and/or systemic coagulopathy [24]. Recognition of PAMPs and DAMPs by monocytes increases the expression of tissue factor (TF) expression, leading to activating the extrinsic coagulation pathway (24). Moreover, the production of neutrophil extracellular traps (NETs), a network composed of acetylated histones and neutrophil-derived DNA, by activated neutrophils, is necessary to trap and kill invading SARS-CoV-2; however, it may promote a severe procoagulant response (24). High production of NETs has been frequently observed in patients with COVID-19, which may be a reason for disease severity [25, 26]. Innate immune response against SARS-CoV-2 has been shown in Fig. 1.

### Adaptive immune response to SARS-CoV-2

The adaptive immune response is the second line for immunological clearance of SARS-CoV-2 through destruction of infected host cells and production of virus-specific antibodies by activated cytotoxic T-cells and B-cells, respectively. Blood lymphopenia is a critical feature in patients with COVID-19, in which B-cells, CD4+ T-cells, and CD8+ T-cells have decreasing amounts (27). Several essential processes are potentially involved in COVID-19-associated lymphopenias such as cytokine-induced pyroptosis and apoptosis of lymphocytes, virus-induced lymphatic organs damage, sequestration of lymphocytes in lungs or other organs, reduced hematopoiesis of bone marrow, direct SARS-CoV-2 infection of T-cells, and MAS-related haemophagocytosis [27–29].

Almost all COVID-19 patients with mild and moderate disease severity demonstrate a robust adaptive immune response by B-cells (antibodies production against S-protein of SARS-CoV-2) and T-cells (against S-protein, nucleoprotein, and membrane protein antigens), which is consistent for several months after primary infection [30]. The number of CD4+ T-cells, CD8+ T-cells, and memory T cells is lower in patients with severe SARS-CoV-2 disease, indicating an inadequate anti-viral response against infection [31]. In addition to the decreased levels of neutralizing antibodies activity, lower levels of IgG have been observed in asymptomatic patients compared to symptomatic patients [32]. Overall, strong adaptive immune responses are associated with milder and moderate disease severity and are essential for optimally controlling viral infection [33]. Adaptive immune response against SARS-CoV-2 has been shown in Fig. 1.

## Lymphocytes B and humoral immunity against SARS-CoV-2

After primary COVID-19 infection, the humoral immune system was activated through stimulation by CD4+ T helper cells or direct interaction with SARS-CoV-2. However, patients indicate a different algorithm during COVID-19 infection [34]. B-cells-secreted antibodies can contribute to the clearance of infected host cells through binding to viral antigens and directing natural killer (NK) cells to kill them via antibody-dependent cell cytotoxicity (ADCC) [35]. Subsequently, memory B-cells are formed, an essential component of long-lasting immunity after viral clearance. Although antibodies are vital components in the release of viral pathogens, some antibodies may lead to abnormal B cell activation and the progression of infection [36].

Since the secretory immunoglobulin A (IgA) protects the mucosal respiratory tract against SARS-CoV-2, it is typically considered the most critical immunoglobulin in neutralizing SARS-CoV-2. In patients with COVID-19 infection, IgA released earlier than other Immunoglobulins, remains longer than IgM, and stimulates the production of pro-inflammatory cytokines such as monocyte chemoattractant proteins (MCPs) and interleukin-6 (IL-6) [37, 38]. In addition, anti-SARS-CoV-2 IgA was identified in patients' saliva and remained for approximately three months after symptoms appearance [39]. High levels of both IgG and IgA are associated with severe COVID-19 infection [40]. Interestingly, although IgA and IgG were detected in breast milk with Covid-19, other body fluids lacked these antibodies. However, seronegative patients mean no immunity [41].

The enhanced expression of several cytokines such as IL-2, IL-6, MCPs, tumor necrosis factor-alpha (TNF-α), granulocyte–macrophage colony-stimulating factor (GM-CSF), macrophage inflammatory protein 1-a (MIP-1a), and IFN-γ-inducible protein 10 (IP-10) as well as several chemokines such as chemokine C–C motif ligand-2 (CCL2) and chemokine C-X-C motif ligands (CXCLs) are observed in patients with SARS-CoV-2 [42–44]. Although B cell responses have been shown to be involved in harming patients with Covid-19, the underlying molecular mechanisms and the precise role of B cells in COVID-19 infection remain unresolved.

## Lymphocytes T and cellular immunity against SARS-CoV-2

Given the high number of infiltrated CD8+ T cells (80%) recruited to the infected area, the researchers believe cellular immunity is the first line of defense against SARS-CoV-2 infection [45]. However, the exhausted infiltrated T cells cause the reduction of non-exhausted CD8+ T-cells in patients with severe COVID-19 [46]. The overexpression of the natural killer group 2 member A (NKG2A) receptor may be one of the leading causes of CD8+ T-cells exhaustion [47]. Previous studies have reported that NKG2A was upregulated in CD8+ T-cells derived from patients with COVID-19 compared to healthy subjects, while it has a decreasing expression pattern in recovered patients [48, 49].

In addition, lymphopenia is another characteristic of cellular immunity in patients with COVID-19 [50]. T-cells are significantly reduced in patients with severe COVID-19, which less than 5% was associated with a high mortality rate [51]. Remarkably, 85% of severe COVID-19 patients with pneumonia indicate lymphopenia [52]. Therefore, the percentage of lymphocytes may be employed as a biomarker to classify infection severity [53]. In addition, the number of cytotoxic NK cells were reduced in patients with COVID-19 and was associated with infection severity [54]. A piece of evidence suggested that SARS-CoV-2 at first caused activation of CD8+ T-cells, then, in turn, led to the exhaustion of CD8+ T-cells. Over-activation of CD8+ T-cells induces uncontrolled cytotoxicity responses, leading to partially tissue damages [55, 56].

Furthermore, SARS-CoV-2 can infect and deplete T cells via binding of S viral spike to CD147 or CD26 on the surface of T cells [57]. Mononuclear macrophage-derived cytokines along with IFN-α and IFN-β cause T cells death through apoptosis [58]. Regulatory T cells (Treg), which play a critical role against respiratory infections, are also affected in patients with COVID-19 [59, 60]. On the other hand, T helper cells (Th1 and Th17) were over-activated, inducing the B cells to produce specific antibodies against SARS-CoV-2 [61, 62].

During viral infection, naive CD8+ T-cells recognize the viral antigens presented on major histocompatibility complex I (MHC I) by their T-cell receptors (TCRs) and then are activated and undergo clonal expansion and differentiation to effector CD8+ T-cells [63]. The effector CD8+ T-cells secrete several cytokines such as TNF and IFN-γ and directly kill pathogenic viruses [64]. A high proportion of effective T cells are directed to apoptosis after the clearance of antigens. Subsequently, a population of memory T cells is stored to fight COVID-19 re-infection in the future. The memory CD4+ T-cells, upon restimulation, trigger B cells, and other immune cells through the production of cytokines, whereas memory cytotoxic T-cells contribute to the destruction of infected host cells during subsequent COVID-19 infection [6, 59, 65]. Unlike memory B cells, memory T cells are preserved for a long time against SARS-CoV-2. Notably, both CD4+ and CD8+ memory T cells are efficient for the immune response for approximately 3 up to 6 years without the presence of any viral antigens [31].

## Exhaustion of immune system during COVID-19

Persistent antigens in chronic viral infection impair the growth of memory CD8+ T cells and impair the function of effective CD8+ T cells; this condition is called CD8+ T cell exhaustion [66]. T cell exhaustion is a powerful mechanism that disrupts the immune response during chronic viral infection [67]. However, the underlying mechanisms that functional cytotoxic lymphocytes exhibited exhausted phenotypes during COVID-19 infection have remained obscure. Recent studies have reported that CD8+ B-cells and CD8+ T-cells are successful in the clearance of mild SARS-CoV-2 infection [46]; whereas further studies on patients with COVID-19 disease have demonstrated that expression of immunoglobulin mucin-3 (TIM-3) and programmed cell death protein-1 (PD-1) upregulated in T cells that are associated with functionally exhaustion of CD8+ T cells [49, 68]. In addition, other pieces of evidence indicated that CD4+ T cells and CD8+ T cells are exhausted in patients with COVID-19 who have reduced expression of IFN-γ and IL-21 [69, 70]. Expression of PD-1 is significantly decreased in patients recovered from COVID-19 compared to severe COVID-19 patients, demonstrating that this exhaustion might be transient [28].

Total NK and CD8+ T cell counts have been shown to be significantly reduced in patients with SARS-CoV-2 infection. NK and CD8+ T cells in patients with persistence COVID-19 infection, which had upregulated levels of NKG2A, exhibited an exhausted phenotype. Notably, in patients recovering from treatment, NK and CD8+ T cells were restored by decreasing NKG2A expression, implying that functional exhaustion of cytotoxic lymphocytes and NK cells is associated with SRAS-CoV-2 infection persistence. Hence, SARS-CoV-2 infection may impair antiviral immunity in a time-dependent manner, leading to depleted immune cells that are dysfunctional against the virus [71, 72]. In both cytotoxic lymphocytes and NK cells of patients with COVID-19 disease, the expression of NKG2A has upregulated as well as expression of IFN-γ, CD107a, granzyme B, and IL-2 is downregulated; this event is consistent with functional exhaustion and disease progression [73–75].

On the other hand, a recent study by Zhang et al. reported that the module score of CD8+ T cells exhaustion was not significantly different between patients with COVID-19 infection, even in patients with severe acute respiratory distress syndrome, and healthy controls [76]. This discrepancy in the results of previous studies may be due to differences in the disease severity criteria and differences in demographic characteristics of the investigated patients in multiple studies.

## Biomarkers of T cell exhaustion in patients with COVID-19

A recent study by Bobcakova et al. reported a significantly lower proportion of CD38+ HLA-DR+ CD8+ cells and a significantly higher proportion of CD38+ CD8+ cells in COVID-19 fatal infections, which is associated with functional exhaustion [77]. Increased expression of PD-1 in the absence of TIM-3 on CD3+ CD4+ and CD3+ CD8+ cells suggests the potential reversibility of immune exhaustion in patients with the most severe COVID-19 infection. In this regard, alone or combined expression level of PD1 on CD4+ T-cells as well as the expression level of CD38 on CD8+ T-cells could be potential biomarkers in the prediction of disease severity and outcome in patients with COVID-19 [46, 77]. In addition, Wilk et al. identified three exhaustion biomarkers include programmed cell death protein 1 (PDCD1), hepatitis A virus cellular receptor 2 (HAVCR2), and lymphocyte activating 3 (LAG3) on NK cells from patients with COVID-19 infection [78].

Previous studies reported that CD38, HLA-DR, and Ki-67 significantly upregulated in CD8+ T cells from patients with severe COVID-19, indicating T cells' activated phenotype [79–81]. However, upregulation of CD39 has been described as an exhaustion phenotype of CD8+ T-cells in patients with severe COVID-19 infection [46]. In addition, several studies reported that the exhaustion phenotype of CD8+ T-cells in patients with severe COVID-19 disease was strongly associated with high expression of several immune checkpoints such as PD-1, TIM-3, LAG-3, CTLA-4, and NKG2A [82, 83]. Increased expression of PD-L1 has been reported in eosinophils and basophils derived from patients with severe COVID-19 infection [84].

A retrospective study by Diao et al. on 522 patients with COVID-19 infection and 40 healthy subjects from Wuhan, China, reported that the clinical severity-dependent and/or age-dependent reduction in the numbers of T cells inversely associated with serum levels of IL-6, IL-10, and TNF [46]. These results demonstrated that signs of infection severity and progress in patients with COVID-19 are associated with increased serum inflammatory cytokine levels due to depletion and exhaustion of T cells [85].

## Immunomodulation approach to boost the immune system

Agents interacting with TLR receptors have been shown to be beneficial in COVID-19 therapy as they are involved in modulating innate immunity. TLRs 3, 7, and 8 are essential PRRs sensing RNA viruses and involved in signalling cascade induced by PAMPs, resulting in the production of interferons necessary for antiviral defense [86, 87]. In this regard, Imiquimod, TLR7 agonist, stimulate specific and nonspecific immune response and cytokine production, thus can be helpful in COVID-19 therapy [88].

Cytokines and chemokines are involved in an inflammatory cascade in which exaggeration may lead to cytokine storms, commonly seen in severe cases of COVID-19. Therefore, modulation of cytokines/chemokines could be a potential therapeutic approach in COVID-19 patients [89]. In this regard, IL-6 receptors blockers, Tocilizumab and sarilumab, as well as TNF-blockers such as etanercept, golimumab, and adalimumab, have been used to treat COVID-19 patients [90, 91]. Ruxolitinib, a JAK-STAT inhibitor that targets IFN-γ, has also treated COVID-19 [92].

CD8+ T cell exhaustion is a characteristic of chronic viral infections such as COVID-19, so reversing its exhaustion by immune checkpoint inhibitors (ICIs) represents a promising treatment strategy [93, 94]. PD-1 and CD39 are two well-defined exhaustion markers expressed on CD8+ T and reflect disease progression. Thus, blocking both CD39/PD-1 pathways simultaneously restored CD8+ T cell function [95, 96]. In this regard, HIV-infected patients treated with PD-1 and CD-39 blockers showed functional T cells instead of exhausted ones [97].

Activating cytotoxic T cells is the most crucial component of an effective immune response against viral infections that apparent infection by killing virus-infected cells. Therefore, the numbers and activated T cells in patients with COVID-19 are essential for successful recovery. Previous studies reported that a high proportion of patients with COVID-19 display a low count of lymphocytes [98, 99]. However, the factors that may reduce exhaustion and induce T cell activation in patients with Covid-19 have been less studied.

Immune exhaustion recovery is a significant objective for developing a novel therapeutic approach for severe viral infections. Lymphopenia is a prominent feature of patients with severe COVID-19 disease, but the functional status of T cells in these patients is unclear. The first study to report that PD1 inhibition was associated with human infections was performed by Walker et al., which showed that suppression of PD1 by monoclonal antibodies increased the number of functional T cells in patients with human immunodeficiency virus (HIV) infection [100]. The upregulation of immune checkpoints (CTLA4, PD-1, and PD-L1) is the typical feature of exhausted CD8+ T cells, decreasing the effector T cells and impairing their ability to increase [101]. The effects of anti-PD-1 and anti-PD-L1 monoclonal antibodies that were previously used as cancer therapy agents have revolutionized, which means that therapeutical inhibition of these pathways may be necessary for preventing and managing various infectious diseases [102].

## Conclusion and a glimpse into the future

**Fig. 2:**
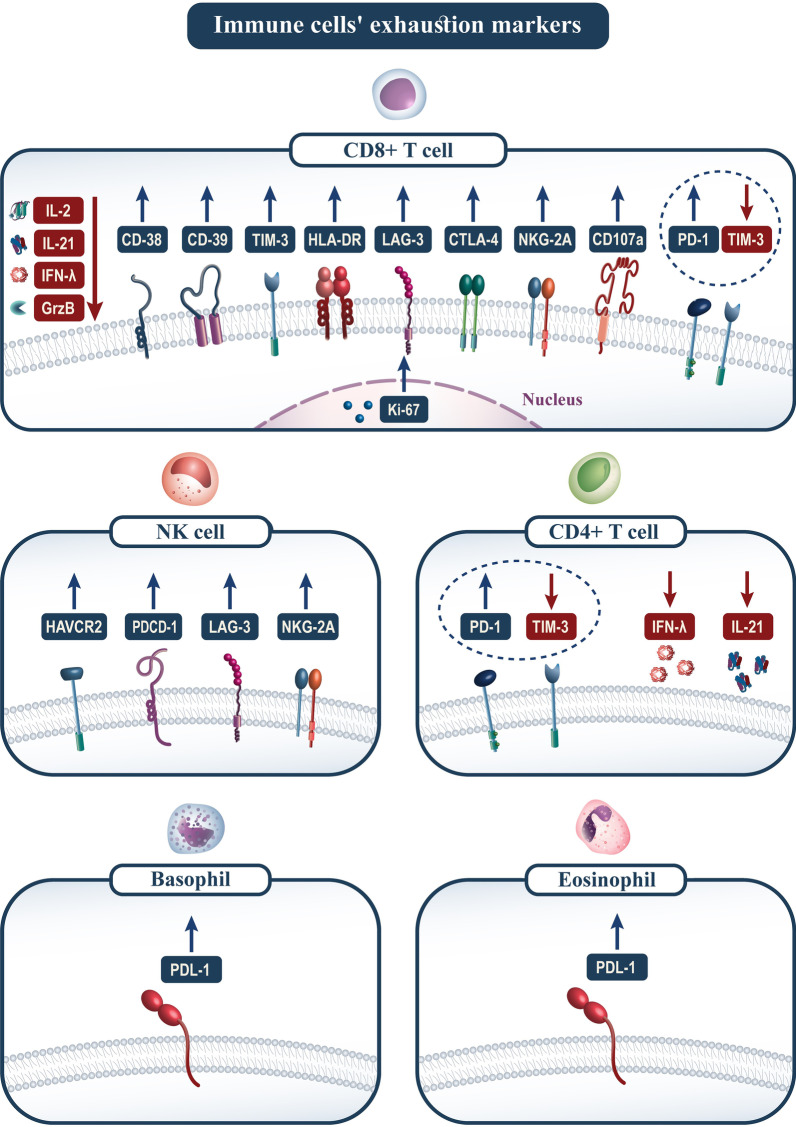
Immune cells' exhaustion markers. The figure shows up-or down-regulation of diverse molecules contributes to the exhaustion of immune systems

With the advent of COVID-19 infection, many studies have enhanced our knowledge of immune responses to coronavirus, such as innate and adaptive immune responses and humoral and cellular immunity. However, the exact mechanism of the immune response and the immune pathology induced by SARS-CoV-2 remain unknown. SARS-CoV-2 compromise the host's immune system in a variety of ways. SARS-CoV-2 specific CD4+ T cells and CD8+ T cells recruited to inflammatory sites are the most critical component of the immune system in the fight against SARS-CoV-2. In this study, markers of immune cell fatigue are shown in Fig. 2. It is believed that the reduction and exhaustion of T cells in patients with COVID-19 infection increase the severity of the disease. However, studies in the field of ICIs blocking exhaustion markers, particularly PD-1 and PDL-1, have shown promising results in reducing the severity of infection. Therefore, further studies on the function and mechanisms of CD8+ T cell exhaustion will help in the management of the COVID-19 epidemic.

## Data Availability

Not applicable.

## Abbreviations

COVID-19: Coronavirus disease
SARS-CoV-2: Coronavirus acute respiratory syndrome
ssRNA: Single-stranded RNA
ACE2: Angiotensin-converting enzyme 2
TMPRSS2: Transmembrane protease serine 2
IFNs: Interferons
DC: Dendritic cells
IgG: Immunoglobulin G
IgM: Immunoglobulin M
CTSL: Cathepsin L
S protein: Spike protein
DAMP: Damage-associated molecular patterns
TLR: Toll-like receptor
PRR: Pattern recognition receptor
IRF: Interferon regulatory factor
NF-κB: Nuclear factor-kappa B
ORF6: Open reading frame 6
TF: Tissue factor
NET: Neutrophil extracellular trap
NK cell: Natural killer cell
MCPs: Monocyte chemoattractant protein
IL-6: Interleukin-6
TNF-α: Tumor necrosis factor-alpha
GM-CSF: Granulocyte–macrophage colony-stimulating factor
MIP-1a: Macrophage inflammatory protein 1-a
CCL2: C–C motif ligand-2
CXCL: C-X-C motif ligand
NKG2A: Natural killer group 2 member A
Treg: Regulatory T cells
MHC I: Major histocompatibility complex I
TCRs: T-cell receptors
TIM-3: T cell immunoglobulin mucin-3
PD-1: Programmed cell death protein-1
ICU: Intensive care unit
HAVCR2: Hepatitis A virus cellular receptor 2
LAG3: Lymphocyte activating 3
CTLA-4: Cytotoxic T-lymphocyte-associated protein 4
HIV: Human immunodeficiency virus
ICI: Immune checkpoint inhibitor

## References

[1] Lai CC, Shih TP, Ko WC, Tang HJ, Hsueh PR (2020). Severe acute respiratory syndrome coronavirus 2 (SARS-CoV-2) and coronavirus disease-2019 (COVID-19): The epidemic and the challenges. Int J Antimicrob Agents.

[2] Hu B, Guo H, Zhou P, Shi ZL (2021). Characteristics of SARS-CoV-2 and COVID-19. Nat Rev Microbiol.

[3] Schultze JL, Aschenbrenner AC (2021). COVID-19 and the human innate immune system. Cell.

[4] Pal M, Berhanu G, Desalegn C, Kandi V (2020). Severe acute respiratory syndrome coronavirus-2 (SARS-CoV-2): an update. Cureus.

[5] Astuti I, Ysrafil (2020). Severe acute respiratory syndrome coronavirus 2 (SARS-CoV-2): an overview of viral structure and host response. Diabetes Metab Syndr.

[6] Shah VK, Firmal P, Alam A, Ganguly D, Chattopadhyay S (1949). Overview of immune response during SARS-CoV-2 infection: lessons from the past. Front Immunol.

[7] Oran DP, Topol EJ (2020). Prevalence of asymptomatic SARS-CoV-2 infection: a narrative review. Ann Intern Med.

[8] Sharma A, Tiwari S, Deb MK, Marty JL (2020). Severe acute respiratory syndrome coronavirus-2 (SARS-CoV-2): a global pandemic and treatment strategies. Int J Antimicrob Agents.

[9] Portela Sousa C, Brites C (2020). Immune response in SARS-CoV-2 infection: the role of interferons type I and type III. Braz J Infect Dis.

[10] Traggiai E, Becker S, Subbarao K, Kolesnikova L, Uematsu Y, Gismondo MR, Murphy BR, Rappuoli R, Lanzavecchia A (2004). An efficient method to make human monoclonal antibodies from memory B cells: potent neutralization of SARS coronavirus. Nat Med.

[11] Lu X, Pan J, Tao J, Guo D (2011). SARS-CoV nucleocapsid protein antagonizes IFN-β response by targeting initial step of IFN-β induction pathway, and its C-terminal region is critical for the antagonism. Virus Genes.

[12] Niu P, Zhang S, Zhou P, Huang B, Deng Y, Qin K, Wang P, Wang W, Wang X, Zhou J (2018). Ultrapotent human neutralizing antibody repertoires against middle east respiratory syndrome coronavirus from a recovered patient. J Infect Dis.

[13] Hoffmann M, Kleine-Weber H, Schroeder S, Krüger N, Herrler T, Erichsen S, Schiergens TS, Herrler G, Wu NH, Nitsche A (2020). SARS-CoV-2 cell entry depends on ACE2 and TMPRSS2 and is blocked by a clinically proven protease inhibitor. Cell.

[14] Mahmoud IS, Jarrar YB, Alshaer W, Ismail S (2020). SARS-CoV-2 entry in host cells-multiple targets for treatment and prevention. Biochimie.

[15] Mogensen TH (2009). Pathogen recognition and inflammatory signaling in innate immune defenses. Clin Microbiol Rev.

[16] Tartey S, Takeuchi O (2017). Pathogen recognition and Toll-like receptor targeted therapeutics in innate immune cells. Int Rev Immunol.

[17] Xia H, Cao Z, Xie X, Zhang X, Chen JY, Wang H, Menachery VD, Rajsbaum R, Shi PY (2020). Evasion of type I interferon by SARS-CoV-2. Cell Rep.

[18] Lei X, Dong X, Ma R, Wang W, Xiao X, Tian Z, Wang C, Wang Y, Li L, Ren L (2020). Activation and evasion of type I interferon responses by SARS-CoV-2. Nat Commun.

[19] Tian W, Zhang N, Jin R, Feng Y, Wang S, Gao S, Gao R, Wu G, Tian D, Tan W (2020). Immune suppression in the early stage of COVID-19 disease. Nat Commun.

[20] Ricklin D, Hajishengallis G, Yang K, Lambris JD (2010). Complement: a key system for immune surveillance and homeostasis. Nat Immunol.

[21] Jin Y, Ji W, Yang H, Chen S, Zhang W, Duan G (2020). Endothelial activation and dysfunction in COVID-19: from basic mechanisms to potential therapeutic approaches. Signal Transduct Target Ther.

[22] Java A, Apicelli AJ, Liszewski MK, Coler-Reilly A, Atkinson JP, Kim AH, Kulkarni HS. The complement system in COVID-19: friend and foe? JCI Insight. 2020;5. 10.1172/jci.insight.140711 PMC7455060 32554923

[23] Li Q, Chen Z (2021). An update: the emerging evidence of complement involvement in COVID-19. Med Microbiol Immunol.

[24] Loo J, Spittle DA, Newnham M (2021). COVID-19, immunothrombosis and venous thromboembolism: biological mechanisms. Thorax.

[25] Ito T (2014). PAMPs and DAMPs as triggers for DIC. J Intensive Care.

[26] Fard MB, Fard SB, Ramazi S, Atashi A, Eslamifar Z (2021). Thrombosis in COVID-19 infection: role of platelet activation-mediated immunity. Thromb J.

[27] García LF (2020). Immune response, inflammation, and the clinical spectrum of COVID-19. Front Immunol.

[28] Vabret N, Britton GJ, Gruber C, Hegde S, Kim J, Kuksin M, Levantovsky R, Malle L, Moreira A, Park MD (2020). Immunology of COVID-19: current state of the science. Immunity.

[29] Sette A, Crotty S (2021). Adaptive immunity to SARS-CoV-2 and COVID-19. Cell.

[30] Zuo J, Dowell AC, Pearce H, Verma K, Long HM, Begum J, Aiano F, Amin-Chowdhury Z, Hoschler K, Brooks T (2021). Robust SARS-CoV-2-specific T cell immunity is maintained at 6 months following primary infection. Nat Immunol.

[31] Le Bert N, Tan AT, Kunasegaran K, Tham CY, Hafezi M, Chia A, Chng MHY, Lin M, Tan N, Linster M (2020). SARS-CoV-2-specific T cell immunity in cases of COVID-19 and SARS, and uninfected controls. Nature.

[32] Long Q-X, Tang X-J, Shi Q-L, Li Q, Deng H-J, Yuan J, Hu J-L, Xu W, Zhang Y, Lv F-J (2020). Clinical and immunological assessment of asymptomatic SARS-CoV-2 infections. Nat Med.

[33] Brodin P (2021). Immune determinants of COVID-19 disease presentation and severity. Nat Med.

[34] Apostolidis SA, Kakara M, Painter MM, Goel RR, Mathew D, Lenzi K, Rezk A, Patterson KR, Espinoza DA, Kadri JC (2021). Cellular and humoral immune responses following SARS-CoV-2 mRNA vaccination in patients with multiple sclerosis on anti-CD20 therapy. Nat Med.

[35] Klasse PJ (2014). Neutralization of virus infectivity by antibodies: old problems in new perspectives. Adv Biol.

[36] Arvin AM, Fink K, Schmid MA, Cathcart A, Spreafico R, Havenar-Daughton C, Lanzavecchia A, Corti D, Virgin HW (2020). A perspective on potential antibody-dependent enhancement of SARS-CoV-2. Nature.

[37] Guo L, Ren L, Yang S, Xiao M, Chang D, Yang F, Dela Cruz CS, Wang Y, Wu C, Xiao Y (2020). Profiling early humoral response to diagnose novel coronavirus disease (COVID-19). Clin Infect Dis.

[38] Padoan A, Sciacovelli L, Basso D, Negrini D, Zuin S, Cosma C, Faggian D, Matricardi P, Plebani M (2020). IgA-Ab response to spike glycoprotein of SARS-CoV-2 in patients with COVID-19: a longitudinal study. Clin Chim Acta.

[39] Huang N, Pérez P, Kato T, Mikami Y, Okuda K, Gilmore RC, Conde CD, Gasmi B, Stein S, Beach M (2021). SARS-CoV-2 infection of the oral cavity and saliva. Nat Med.

[40] Zervou FN, Louie P, Stachel A, Zacharioudakis IM, Ortiz-Mendez Y, Thomas K, Aguero-Rosenfeld ME (2021). SARS-CoV-2 antibodies: IgA correlates with severity of disease in early COVID-19 infection. J Med Virol.

[41] Dong Y, Chi X, Hai H, Sun L, Zhang M, Xie WF, Chen W (2020). Antibodies in the breast milk of a maternal woman with COVID-19. Emerg Microbes Infect.

[42] Coperchini F, Chiovato L, Rotondi M (2021). Interleukin-6, CXCL10 and infiltrating macrophages in COVID-19-related cytokine storm: not one for all but all for one!. Front Immunol.

[43] Chen S, Yang L, Nilsson-Payant B, Han Y, Jaffré F, Zhu J, Wang P, Zhang T, Redmond D, Houghton S, et al. SARS-CoV-2 infected cardiomyocytes recruit monocytes by secreting CCL2. Res Sq. 2020.

[44] Santa Cruz A, Mendes-Frias A, Oliveira AI, Dias L, Matos AR, Carvalho A, Capela C, Pedrosa J, Castro AG, Silvestre R (2021). Interleukin-6 is a biomarker for the development of fatal severe acute respiratory syndrome coronavirus 2 pneumonia. Front Immunol.

[45] Urra JM, Cabrera CM, Porras L, Ródenas I (2020). Selective CD8 cell reduction by SARS-CoV-2 is associated with a worse prognosis and systemic inflammation in COVID-19 patients. Clin Immunol.

[46] Diao B, Wang C, Tan Y, Chen X, Liu Y, Ning L, Chen L, Li M, Liu Y, Wang G (2020). Reduction and functional exhaustion of T cells in patients with coronavirus disease 2019 (COVID-19). Front Immunol.

[47] Yaqinuddin A, Kashir J (2020). Innate immunity in COVID-19 patients mediated by NKG2A receptors, and potential treatment using Monalizumab, Cholroquine, and antiviral agents. Med Hypotheses.

[48] Yasin MM, Shehata IH, Elsheikh NG, Elsayed MS (2021). Expression of NKG2A inhibitory receptor on cytotoxic lymphocytes as an indicator of severity in Corona Virus Disease 2019 (COVID-19) patients. Egypt J Immunol.

[49] Chen Z, John WE (2020). T cell responses in patients with COVID-19. Nat Rev Immunol.

[50] Zhou X, Ye Q (2021). Cellular immune response to COVID-19 and potential immune modulators. Front Immunol.

[51] Bange EM, Han NA, Wileyto P, Kim JY, Gouma S, Robinson J, Greenplate AR, Hwee MA, Porterfield F, Owoyemi O (2021). CD8(+) T cells contribute to survival in patients with COVID-19 and hematologic cancer. Nat Med.

[52] Bermejo-Martin JF, Almansa R, Menéndez R, Mendez R, Kelvin DJ, Torres A (2020). Lymphopenic community acquired pneumonia as signature of severe COVID-19 infection. J Infect.

[53] Tan L, Wang Q, Zhang D, Ding J, Huang Q, Tang YQ, Wang Q, Miao H (2020). Lymphopenia predicts disease severity of COVID-19: a descriptive and predictive study. Signal Transduct Target Ther.

[54] Ahmed F, Jo DH, Lee SH (2020). Can natural killer cells be a principal player in anti-SARS-CoV-2 immunity?. Front Immunol.

[55] De Biasi S, Meschiari M, Gibellini L, Bellinazzi C, Borella R, Fidanza L, Gozzi L, Iannone A, Tartaro DL, Mattioli M (2020). Marked T cell activation, senescence, exhaustion and skewing towards TH17 in patients with COVID-19 pneumonia. Nat Commun.

[56] Bertoletti A, Le Bert N, Qui M, Tan AT (2021). SARS-CoV-2-specific T cells in infection and vaccination. Cell Mol Immunol.

[57] Wang K, Chen W, Zhang Z, Deng Y, Lian JQ, Du P, Wei D, Zhang Y, Sun XX, Gong L (2020). CD147-spike protein is a novel route for SARS-CoV-2 infection to host cells. Signal Transduct Target Ther.

[58] Ye Q, Wang B, Mao J (2020). The pathogenesis and treatment of the `Cytokine Storm' in COVID-19. J Infect.

[59] Peng X, Ouyang J, Isnard S, Lin J, Fombuena B, Zhu B, Routy JP (2020). Sharing CD4+ T cell loss: when COVID-19 and HIV collide on immune system. Front Immunol.

[60] Wang Y, Zheng J, Islam MS, Yang Y, Hu Y, Chen X (2021). The role of CD4(+)FoxP3(+) regulatory T cells in the immunopathogenesis of COVID-19: implications for treatment. Int J Biol Sci.

[61] Costela-Ruiz VJ, Illescas-Montes R, Puerta-Puerta JM, Ruiz C, Melguizo-Rodríguez L (2020). SARS-CoV-2 infection: the role of cytokines in COVID-19 disease. Cytokine Growth Factor Rev.

[62] Apostolidis SA, Kakara M, Painter MM, Goel RR, Mathew D, Lenzi K, Rezk A, Patterson KR, Espinoza DA, Kadri JC (2021). Cellular and humoral immune responses following SARS-CoV-2 mRNA vaccination in patients with multiple sclerosis on anti-CD20 therapy. Nat Med.

[63] Sigal LJ (2016). Activation of CD8 T lymphocytes during viral infections. Encycl Immunobiol.

[64] Cox MA, Kahan SM, Zajac AJ (2013). Anti-viral CD8 T cells and the cytokines that they love. Virology.

[65] Wherry EJ, Kurachi M (2015). Molecular and cellular insights into T cell exhaustion. Nat Rev Immunol.

[66] Yi JS, Cox MA, Zajac AJ (2010). T-cell exhaustion: characteristics, causes and conversion. Immunology.

[67] Saeidi A, Zandi K, Cheok YY, Saeidi H, Wong WF, Lee CYQ, Cheong HC, Yong YK, Larsson M, Shankar EM (2018). T-cell exhaustion in chronic infections: reversing the state of exhaustion and reinvigorating optimal protective immune responses. Front Immunol.

[68] Herrmann M, Schulte S, Wildner NH, Wittner M, Brehm TT, Ramharter M, Woost R, Lohse AW, Jacobs T, Schulze zur Wiesch J (1870). Analysis of co-inhibitory receptor expression in COVID-19 infection compared to acute plasmodium falciparum malaria: lAG-3 and TIM-3 correlate with t cell activation and course of disease. Front Immunol.

[69] de Candia P, Prattichizzo F, Garavelli S, Matarese G (2021). T cells: warriors of SARS-CoV-2 infection. Trends Immunol.

[70] Bange EM, Han NA, Wileyto P, Kim JY, Gouma S, Robinson J, Greenplate AR, Hwee MA, Porterfield F, Owoyemi O (2021). CD8+ T cells contribute to survival in patients with COVID-19 and hematologic cancer. Nat Med.

[71] van Eeden C, Khan L, Osman MS, Cohen Tervaert JW (2020). Natural killer cell dysfunction and its role in COVID-19. Int J Mol Sci.

[72] Antonioli L, Fornai M, Pellegrini C, Blandizzi C (2020). NKG2A and COVID-19: another brick in the wall. Cell Mol Immunol.

[73] Taefehshokr N, Taefehshokr S, Hemmat N, Heit B (2020). Covid-19: perspectives on innate immune evasion. Front Immunol.

[74] Zheng M, Gao Y, Wang G, Song G, Liu S, Sun D, Xu Y, Tian Z (2020). Functional exhaustion of antiviral lymphocytes in COVID-19 patients. Cell Mol Immunol.

[75] Witkowski M, Tizian C, Ferreira-Gomes M, Niemeyer D, Jones TC, Heinrich F, Frischbutter S, Angermair S, Hohnstein T, Mattiola I (2021). Untimely TGFβ responses in COVID-19 limit antiviral functions of NK cells. Nature.

[76] Zhang JY, Wang XM, Xing X, Xu Z, Zhang C, Song JW, Fan X, Xia P, Fu JL, Wang SY (2020). Single-cell landscape of immunological responses in patients with COVID-19. Nat Immunol.

[77] Bobcakova A, Petriskova J, Vysehradsky R, Kocan I, Kapustova L, Barnova M, Diamant Z, Jesenak M (2021). Immune profile in patients with COVID-19: lymphocytes exhaustion markers in relationship to clinical outcome. Front Cell Infect Microbiol.

[78] Wilk AJ, Rustagi A, Zhao NQ, Roque J, Martínez-Colón GJ, McKechnie JL, Ivison GT, Ranganath T, Vergara R, Hollis T (2020). A single-cell atlas of the peripheral immune response in patients with severe COVID-19. Nat Med.

[79] Sekine T, Perez-Potti A, Rivera-Ballesteros O, Strålin K, Gorin JB, Olsson A, Llewellyn-Lacey S, Kamal H, Bogdanovic G, Muschiol S (2020). Robust T cell immunity in convalescent individuals with asymptomatic or mild COVID-19. Cell.

[80] Kuri-Cervantes L, Pampena MB, Meng W, Rosenfeld AM, Ittner CAG, Weisman AR, Agyekum RS, Mathew D, Baxter AE, Vella LA (2020). Comprehensive mapping of immune perturbations associated with severe COVID-19. Sci Immunol.

[81] Szabo PA, Dogra P, Gray JI, Wells SB, Connors TJ, Weisberg SP, Krupska I, Matsumoto R, Poon MML, Idzikowski E (2021). Longitudinal profiling of respiratory and systemic immune responses reveals myeloid cell-driven lung inflammation in severe COVID-19. Immunity.

[82] Mudd PA, Crawford JC, Turner JS, Souquette A, Reynolds D, Bender D, Bosanquet JP, Anand NJ, Striker DA, Martin RS (2020). Distinct inflammatory profiles distinguish COVID-19 from influenza with limited contributions from cytokine storm. Sci Adv.

[83] Paolini A, Borella R, De Biasi S, Neroni A, Mattioli M, Lo Tartaro D, Simonini C, Franceschini L, Cicco G, Piparo AM (2021). Cell death in coronavirus infections: uncovering its role during COVID-19. Cells.

[84] Vitte J, Diallo AB, Boumaza A, Lopez A, Michel M, Allardet-Servent J, Mezouar S, Sereme Y, Busnel JM, Miloud T (2020). A granulocytic signature identifies COVID-19 and its severity. J Infect Dis.

[85] Vardhana SA, Wolchok JD. The many faces of the anti-COVID immune response. J Exp Med. 2020;217. 10.1084/jem.20200678 PMC7191310 32353870

[86] Patra R, Chandra Das N, Mukherjee S (2021). Targeting human TLRs to combat COVID-19: a solution?. J Med Virol.

[87] Poulas K, Farsalinos K, Zanidis C (2020). Activation of TLR7 and innate immunity as an efficient method against COVID-19 pandemic: imiquimod as a potential therapy. Front Immunol.

[88] Angelopoulou A, Alexandris N, Konstantinou E, Mesiakaris K, Zanidis C, Farsalinos K, Poulas K (2020). Imiquimod-a toll like receptor 7 agonist-is an ideal option for management of COVID 19. Environ Res.

[89] Iqbal Yatoo M, Hamid Z, Rather I, Nazir QUA, Bhat RA, Ul Haq A, Magray SN, Haq Z, Sah R, Tiwari R (2021). Immunotherapies and immunomodulatory approaches in clinical trials: a mini review. Hum Vaccin Immunother.

[90] Feldmann M, Maini RN, Woody JN, Holgate ST, Winter G, Rowland M, Richards D, Hussell T (2020). Trials of anti-tumour necrosis factor therapy for COVID-19 are urgently needed. Lancet.

[91] Rahmati M, Moosavi MA (2020). Cytokine-targeted therapy in severely ill COVID-19 patients: options and cautions. EJMO.

[92] Capochiani E, Frediani B, Iervasi G, Paolicchi A, Sani S, Roncucci P, Cuccaro A, Franchi F, Simonetti F, Carrara D (2020). Ruxolitinib rapidly reduces acute respiratory distress syndrome in COVID-19 disease. Analysis of data collection from RESPIRE protocol. Front Med (Lausanne).

[93] Gupta PK, Godec J, Wolski D, Adland E, Yates K, Pauken KE, Cosgrove C, Ledderose C, Junger WG, Robson SC (2015). CD39 expression identifies terminally exhausted CD8+ T cells. PLoS Pathog.

[94] Trautmann L, Janbazian L, Chomont N, Said EA, Gimmig S, Bessette B, Boulassel MR, Delwart E, Sepulveda H, Balderas RS (2006). Upregulation of PD-1 expression on HIV-specific CD8+ T cells leads to reversible immune dysfunction. Nat Med.

[95] Zahm CD, Colluru VT, McIlwain SJ, Ong IM, McNeel DG (2018). TLR stimulation during T-cell activation lowers PD-1 expression on CD8+ T cells. Cancer Immunol Res.

[96] Xiao J, Liu X, Yu X, Dong Y, Wang X, Leng J, Yang R, Wang Y (2016). Upregulated PD-1 on CD8(+)T cells is positively correlated with activation of T cells during HIV-1 infection. Xi Bao Yu Fen Zi Mian Yi Xue Za Zhi.

[97] Li J, Huang HH, Tu B, Zhou MJ, Hu W, Fu YL, Li XY, Yang T, Song JW, Fan X (2021). Reversal of the CD8(+) T-cell exhaustion induced by chronic HIV-1 infection through combined blockade of the adenosine and PD-1 pathways. Front Immunol.

[98] Huang C, Wang Y, Li X, Ren L, Zhao J, Hu Y, Zhang L, Fan G, Xu J, Gu X (2020). Clinical features of patients infected with 2019 novel coronavirus in Wuhan, China. Lancet.

[99] Chen N, Zhou M, Dong X, Qu J, Gong F, Han Y, Qiu Y, Wang J, Liu Y, Wei Y (2020). Epidemiological and clinical characteristics of 99 cases of 2019 novel coronavirus pneumonia in Wuhan, China: a descriptive study. Lancet.

[100] Kaufmann DE, Walker BD (2009). PD-1 and CTLA-4 inhibitory cosignaling pathways in HIV infection and the potential for therapeutic intervention. J Immunol.

[101] Isazadeh A, Hajazimian S, Garshasbi H, Shadman B, Baghbanzadeh A, Chavoshi R, Taefehshokr S, Farhoudi Sefidan Jadid M, Hajiasgharzadeh K, Baradaran B (2021). Resistance mechanisms to immune checkpoints blockade by monoclonal antibody drugs in cancer immunotherapy: focus on myeloma. J Cell Physiol.

[102] Vivarelli S, Falzone L, Torino F, Scandurra G, Russo G, Bordonaro R, Pappalardo F, Spandidos DA, Raciti G, Libra M (2021). Immune-checkpoint inhibitors from cancer to COVID-19: a promising avenue for the treatment of patients with COVID-19 (review). Int J Oncol.

